# Parental stress, mental health, and child traits in Italian mothers and fathers of autistic children

**DOI:** 10.3389/fpsyg.2025.1593255

**Published:** 2025-07-02

**Authors:** Silvia Perzolli, Giulio Bertamini, Paola Venuti, Arianna Bentenuto

**Affiliations:** ^1^Laboratory of Observation, Diagnosis and Education (ODFLab), Department of Psychology and Cognitive Science, University of Trento, Rovereto, Italy; ^2^Department of Child and Adolescent Psychiatry, Pitie-Salpetriere University Hospital, Sorbonne University, Paris, France

**Keywords:** autism, mothers and fathers, parent-reported child behaviors, stress, mental health

## Abstract

**Introduction:**

Several studies have investigated differences between mothers' and fathers' stress and mental health in the context of autistic children parenting. However, fewer have examined differences in parent-reported perceptions of their children's behaviors, symptom severity, and their associations with parental variables. This study aimed to compare parental stress (Parental Stress Index—Short From), mental health (Symptom Checklist–90), and parent-reported perceptions of child behaviors (Child Behavior Checklist—CBCL) and symptom severity (Social Responsiveness Scale—SRS). In Italian mothers and fathers of autistic children while exploring how parental wellbeing relates to both parent-reported and clinically assessed child characteristics.

**Methods:**

A total of 102 parents (51 mothers and 51 fathers) of autistic children aged 4–19 years completed questionnaires assessing stress, mental health, children's behavioral traits and symptom severity. Standardized clinical tests directly measured children's cognitive functioning (IQ) and symptom severity. Group differences between mothers and fathers were analyzed using inferential tests, while Linear Mixed Models were employed to examine the effect of parent, parental stress and mental health on parent-reported perceptions of children's behaviors and symptom severity, as well as clinically measured children's cognitive functioning and symptoms severity.

**Results:**

Mothers and fathers reported similar stress levels but mothers showed higher levels of mental health symptoms and perceived their children as having more severe internalizing and externalizing behaviors and more severe symptomatology compared to fathers. Parental mental health was significantly associated with parent-reported child internalizing behaviors, while parental stress was linked to externalizing behaviors and parent-reported autism severity. No effects emerged for child cognitive functioning, and the model on clinician-rated autism severity failed to converge.

**Discussion:**

These findings emphasize the interconnected nature of parental wellbeing, parent-reported child characteristics, and clinically assessed child traits. Recognizing these links may inform more effective, targeted support strategies for families raising autistic children.

## 1 Introduction

Core challenges associated with autism, such as difficulties in communication, social interaction, and behavioral flexibility, may influence the child's ability to engage with significant others, including parents (Crowell et al., 2019). These challenges often transform everyday interactions into complex and demanding situations, making it difficult for parents to connect with their children through joyful and shared activities. This dynamic may constitute a significant challenge for parents, contributing to elevated stress levels and influencing the overall emotional climate in which the child develops (Ting and Weiss, 2017).

In recent years, there has been a growing body of literature focusing on stress and mental health of parents raising autistic children, often compared with parents of typically developing children (Estes et al., 2013; Baker-Ericzén et al., 2005; Hoffman et al., 2009) or children with other diagnoses (Davies et al., 2021; Barroso et al., 2018). These findings underscore the unique and multifaceted challenges faced by parents of autistic children. Such difficulties can affect parent-child interactions and may contribute to cycles of maladaptive responses, impacting both the child's development and parental wellbeing (Piro-Gambetti et al., 2024). As a result, families may experience heightened levels of parental stress, emotional exhaustion, and difficulties in fostering healthy relational dynamics within the family unit (Davis and Carter, 2008; Hoffman et al., 2009; Baker-Ericzén et al., 2005). Therefore, understanding and addressing the unique experiences and needs of parents of autistic children in both research and clinical settings is a cornerstone for promoting more effective interventions and fostering resilience in these families. Moreover, a notable gap in research literature still exists: while the maternal perspective is often prioritized, there is limited research focusing on fathers, despite their essential role in supporting child development and the family system (Hirschler-Guttenberg et al., 2015; Rankin et al., 2019).

### 1.1 Parental stress

Stress among parents of autistic children has been extensively studied, but results are often inconsistent. Some research revealed differences between mothers and fathers (Vasilopoulou and Nisbet, 2016; Pisula, 2011). However, other studies found no differences between the two caregivers (Davis and Carter, 2008; Ozturk et al., 2014). Child-related factors such as symptom severity, social difficulties, adaptive functioning, and emotional difficulties significantly predicted maternal stress (Mello et al., 2022). In contrast, paternal stress seemed to be less influenced by these factors (Li et al., 2022).

Nonetheless, high levels of stress have been found to be strong predictors of anxiety and depression in both mothers and fathers, indicating a need for broader support (Li et al., 2022). Parental stress was also strongly associated with parental perceptions of child characteristics, such as externalizing behavioral problems, that can in turn amplify parental stress (Lin et al., 2021). However, their relationship may not always be reciprocal, as behavioral problems in children may not necessarily predict parental stress. In fact, beyond the critical period of diagnosis, studies suggested higher stress in parents of younger children (Barker et al., 2011; Fitzgerald et al., 2002; Lounds et al., 2007; Smith et al., 2008), increasing stress with age (e.g., Konstantareas and Homatidis, 1989; Tehee et al., 2009), as well as stable levels of stress over time (Lecavalier et al., 2006; McStay et al., 2014). This evidence highlights the importance of deepening our understanding of the specific associations between these dimensions.

Systematic reviews (e.g., Enea and Rusu, 2020) have identified numerous factors influencing parenting stress, categorized into child-related and parent-related variables. Child-related factors include problematic behaviors (Yorke et al., 2018; Estes et al., 2013), the severity of autistic symptoms (Pastor-Cerezuela et al., 2016; Machado Junior et al., 2016), and adaptive or maladaptive functioning (Operto et al., 2021). Parent-related factors include self-efficacy (Reed et al., 2017; Stephenson et al., 2023), resilience (Faso et al., 2013), co-parenting dynamics, and parenting styles. Research consistently showed that greater severity, reduced adaptive functioning, and heightened behavioral problems (internalizing and externalizing) in children are associated with higher stress levels among parents (Operto et al., 2021). A recent study (Rodriguez et al., 2019) investigated the bidirectional relationship between parents' stress and child functioning and found that parenting stress positively predicted later increases in internalizing behaviors in autistic children, but not vice versa. Effects were more prominent in mothers, also in line with previous literature (Rodriguez et al., 2019), highlighting that high parenting stress in mothers may lead to increases in the child with ASD's internalized behavior problems. Parental stress led to increases in child externalizing behavior problems in mothers and fathers of autistic children. This pattern suggests that a context of high parental stress may not only lead to child internalizing behavior problems, such as withdrawal and avoidance, but also to child externalizing behavior problems, such as aggression and impulsivity. Overall, these findings highlight the importance of addressing both child-related and parent-related factors in interventions, as they significantly influence parental stress, mental health challenges, and family dynamics (Yorke et al., 2018; Lin et al., 2021).

### 1.2 Mental health

While much research has focused on parental stress in the context of autism, less attention has been given to other clinical domains of parenting, such as mental health, which reflects psychological wellbeing (Schnabel et al., 2020). Research has consistently shown that parents of autistic children tend to experience higher levels of depression and anxiety compared to parents of typically developing children (Alibekova et al., 2022). Parents of autistic children also reported higher mental health concerns than those with other difficulties, such as intellectual disabilities (Gau et al., 2012; Karst and Van Hecke, 2012). Notably, mothers tended to exhibit higher levels of depression and anxiety (Davis and Carter, 2008; Ozturk et al., 2014; Demšar and Bakracevic, 2023), and these mental health issues were correlated with the severity of their children's behavioral symptoms (Machado Junior et al., 2016). One possible explanation for this is that mothers are often involved in caregiving and assume more managerial responsibilities, and for this, they can experience more psychological symptoms (e.g., depression) than fathers (Crnic and Low, 2002).

While much of the existing literature highlights the stress and psychological burden that can arise from raising a child with autism, it is important to acknowledge that the mechanisms underlying parental mental health are likely more complex. Emerging research suggests that shared genetic factors, such as elevated autistic or neurodivergent traits in parents themselves, may contribute to increased vulnerability to mental health difficulties (Schnabel et al., 2020). Additionally, environmental stressors disproportionately experienced by neurodivergent individuals—including social exclusion, economic instability, and employment barriers—may further exacerbate psychological strain (Yorke et al., 2018; Alibekova et al., 2022). Considering these intersecting influences offers a more nuanced understanding of the pathways linking child autism and parental wellbeing.

Recent studies have also highlighted a positive correlation between autistic symptoms in children and depressive and anxious symptoms in their parents, with the relationship mediated by concerns about the future, parenting stress, marital conflict, and economic pressure on the family (Chan et al., 2018). Furthermore, research suggests that the severity of children's behavioral problems is related to higher levels of anxiety and depression among mothers (Barker et al., 2011; Bitsika and Sharpley, 2004; Wiggins et al., 2019). Jones et al. (2013) suggested that behavioral problems and adaptive difficulties in autistic children are more strongly associated with maternal anxiety than paternal anxiety. The probability of experiencing depression is also higher in parents as their child ages or when a family has more than one child with autism (Cohrs and Leslie, 2017). Collectively, these findings underscore the significant mental health challenges faced by parents of autistic children and highlight the need for targeted interventions to address parents' wellbeing and children's developmental needs. In line with this, inclusion of fathers in intervention programs and design plays a crucial role. In fact, despite growing recognition of the father's role in child development, especially in the context of autism, research continues to underrepresent paternal perspectives. This gap limits our understanding of fathers' unique contributions and stressors experienced by fathers, which may differ significantly from mothers' emotional expression, co-regulation strategies, and caregiving patterns (Davis and Carter, 2008; Patel et al., 2022). Moreover, excluding fathers may obscure important relational dynamics within the family system and hinder the development of support strategies that are inclusive of all caregiving roles. Recent work has emphasized that paternal wellbeing and involvement are crucial for child adjustment and maternal mental health, suggesting that a more integrative approach to family research is needed (Fisher and Glangeaud-Freudenthal, 2023). Although several studies focused on stress and mental health, results are still inconsistent (Schnabel et al., 2020 for a review). Also, only a few studies had a balanced sample of mothers and fathers, underscoring the need to conduct more research including fathers with paired samples to perform targeted comparisons. Furthermore, emerging research supports a transactional perspective in which parental wellbeing and child behaviors are interdependent and mutually reinforcing over time (Rodriguez et al., 2019). For example, child externalizing behaviors can increase parental stress, while parental internalizing symptoms may sensitize caregivers to children's internal struggles. Understanding these reciprocal influences is crucial for designing targeted interventions that address the needs of both parents and children within the family system.

### 1.3 Parent-reported child behaviors

In line with previous research on differences between mothers' and fathers' reports of child internalizing and externalizing behaviors (Rodriguez et al., 2019) and autism symptomatology (Rodriguez et al., 2019; Falk et al., 2014), the first aim of this study was to compare parental mental health, stress, and parent-reported perceptions of their children's behaviors and symptom severity in an Italian sample of parents and their autistic children.

Second, although previous research studied bidirectional associations between stress and parent reported child behaviors and symptoms (Rodriguez et al., 2019), fewer studies focused on the association between parents' mental health and their parent-reported representations. Therefore, we aimed at exploring potential associations between parents' traits, their parent-reported characteristics of internalizing and externalizing behaviors, and the severity of the perceived symptoms. In addition, despite recent strengths in involving fathers in the context of autism research, there is still limited empirical focus on fathers, especially in efforts to disentangle the specific role of fathers from that of mothers. To our knowledge, no previous research including all these dimensions has been conducted within an Italian sample.

Based on the above-mentioned findings, we addressed the following hypotheses:

1. In line with previous studies (Enea and Rusu, 2020; Mello et al., 2022; Schnabel et al., 2020; Rattaz et al., 2023), we expected mothers to report higher stress and mental health difficulties than fathers in our sample.
2. In line with previous research that investigated the differences between mothers' and fathers' reports of child internalizing and externalizing behaviors (Rodriguez et al., 2019), we hypothesized no differences in the perceptions of their children's behaviors. However, considering previous research on parental perceptions of autistic children's symptomatology (Rodriguez et al., 2019; Falk et al., 2014), we hypothesized that mothers may report higher levels of perceived severity.
3. While previous studies support an association between parental stress and certain behavioral features of the child (see the above literature), fewer empirical findings are available on the link between parent-reported symptom representations in the context of the fathers' literature. In line with this, we assessed associations between parental stress and mental health and the child's perceived behaviors and severity.
4. Finally, we aimed to investigate whether parental stress and mental health are associated with clinician-rated measures of child cognitive function and symptom severity.

## 2 Materials and methods

### 2.1 Participants and procedure

The sample included *n* = 112 parents (*n* = 56 mothers and *n* = 56 fathers) of autistic individuals aged between 4 and 19 (mean chronological age = 110.77 months; *SD* = 48.93; mean mental age = 110.96 months; *SD* = 70.94). Data were collected at the Laboratory of Observation, Diagnosis, and Education (ODFLab), a clinical and research center of the Department of Psychology of the University of Trento, Italy. Participants were recruited on a voluntary basis. Families independently contacted the center to request a developmental assessment or interventions for their children. Recruitment materials, including flyers in the clinic waiting area and announcements shared via the lab's website, newsletter, and social media, advertised the research opportunity. Interested parents were subsequently invited to a dedicated meeting with a member of the research team, during which study aims, procedures, and ethical safeguards were explained. Written informed consent was obtained from both parents when joint custody applied. Participants were informed they could withdraw at any point, and in such cases, collected data would be excluded from the study.

Children underwent a complete clinical assessment with gold-standard procedures for diagnosing neurodevelopmental conditions. The diagnosis of Autism Spectrum Disorder was confirmed by following the DSM-5 criteria (American Psychiatric Association, 2013) and through the administration of the Autism Diagnostic Observation Schedule-2 (ADOS-2) by trained clinicians (Lord et al., 2012). Parents completed questionnaires for parental stress, mental health, behavioral and emotional problems of their children, and their children's symptomatology. All participants signed a written consent to participate in the study. The procedures of this study followed the last version of the Declaration of Helsinki (General Assembly of the World Medical Association, 2014) and were approved by the Research Ethics Board of the University of Trento (Protocol Number 2020-042). The initial sample of this study included a significantly larger number of participants. However, attrition occurred due to the rigorous application of inclusion criteria. Specifically, participants were included only if they met the following criteria: (1) a confirmed diagnosis of Autism Spectrum Disorder (ASD) through standardized diagnostic assessments; (2) an age range between 4 and 19 years; (3) at least one cognitive functioning assessment available for the child; (4) participation of both parents in the entire assessment process, with the completion of all measures related to their mental health and their parent-reported representations of the child. Participants were automatically excluded from the study if any of these measures were missing. This process ensured the inclusion of complete and homogeneous data, which was essential for the validity of the analyses conducted, resulting in *N* = 51 paired dyads {mean child CA = 116.49 months (47.61); range = [49, 213]; mean child MA = 118.10 months (70.64); range = [22, 275]; mean CA Mothers = 43.42 years (6.25); range = [31, 58]; mean CA Fathers = 47.06 years (7.60); range = [34, 73]}. The average ADOS-2 Calibrated Score was 6.47 (1.37), ranging from 3 to 9. As for the Cognitive Profile, Children's average IQ was 87.55 (31.91), ranging from 20 to 135.

### 2.2 Measures

#### 2.2.1 Individual parental measures

##### 2.2.1.1 Parental stress index—Short form

The Parental Stress Index—Short Form (PSI-SF; Abidin, 1990) is a self-report questionnaire to identify early signs of stress in the parent-child relationship. The tool comprises 36 items rated on a 5-point Likert scale (ranging from agreement to disagreement) and evaluates three key dimensions of parental stress:

- Parental Distress (PD): Assesses the level of stress parents experience in their parenting role due to individual factors independent of the child.
- Dysfunctional Parent-Child Interaction (P-CDI): Evaluates the parent's perception of the relationship with the child.
- Difficult Child (DC): Examines characteristics of the child's behavior and the parent's perception of raising a challenging child.

Finally, a Total Stress Index (ST) is calculated as the sum of the three primary domains. We considered percentile scores as provided by the standardized scoring procedure of the instrument, rather than the raw scores. Scores between 75 and 90 are considered clinically relevant.

##### 2.2.1.2 Symptom checklist–90 revised

The Symptom Checklist-90-Revised (SCL-90-R; Derogatis and Savitz, 1999) is a 90-item self-report questionnaire that evaluates a broad range of psychological symptoms in both clinical and non-clinical populations. It captures the psychological symptom state at a specific time and aids in identifying potential issues such as anxiety or depression, warranting further assessment. Each item is rated on a 5-point scale (0–4) from “not at all” to “very much”, and results are interpreted across nine symptom dimensions and three global indices. The symptom dimensions are *Somatization; Obsessive-Compulsive; Interpersonal Sensitivity; Depression; Anxiety; Hostility; Phobic Anxiety; Paranoid Ideation; Psychoticism*. The global indices are:

- *Global Severity Index (GSI)*: Overall severity of psychological distress, combining symptom number and intensity.
- *Positive Symptom Total (PST)*: Total number of symptoms reported.
- *Positive Symptom Distress Index (PSDI)*: Average distress per symptom, reflecting response style and symptom intensity.

Scores exceeding “1” on global indices indicate clinical relevance, while scores above “2” suggest severe symptoms.

#### 2.2.2 Parent-reported child measure

##### 2.2.2.1 Child-behavior checklist

The Child Behavior Checklist (CBCL; Achenbach and Rescorla, 2000, 2001) is a parent-reported questionnaire designed to evaluate children's competencies and behavioral problems. The assessment of problematic behaviors is through items rated by parents on a 3-point Likert scale (0–2), indicating the frequency of specific behaviors over the past 6 months. Several scales are organized into two broad categories: the Internalizing Problems scale (comprising Withdrawal, Somatic Complaints, and Anxiety/Depression) and the Externalizing Problems scale (including Rule-Breaking and Aggressive Behavior). T-scores above 70 are clinically significant, whereas scores between 65 and 69 are borderline and they warrant attention.

##### 2.2.2.2 Social responsiveness scale

The Social Responsiveness Scale—Second Edition (SRS-2; Constantino and Gruber, 2012) is a self-report questionnaire designed to assess the presence and severity of social challenges in ASD. It comprises 65 items and is suitable for parents or caregivers of individuals aged 2.5 years through adulthood.

The SRS-2 evaluates two core symptom domains defined by the DSM-5. In the area of social interaction and communication, it includes the scales of Social Awareness (AWR), Social Cognition (COG), Social Communication (COM), and Social Motivation (MOT). Additionally, it assesses Restricted and Repetitive Behaviors and Interests (RRB) as a separate domain. T-scores above 75 indicate a severe level of autistic symptomatology. Scores between 75 and 66 indicate moderate levels of autistic symptomatology, whereas scores between 60 and 65 indicate mild levels of symptomatology. In general, T-scores below 60 are not clinically significant.

#### 2.2.3 Clinician rated child assessments

##### 2.2.3.1 Autism diagnostic observation schedule 2—ADOS-2

In this study, the Autism Diagnostic Observation Schedule-2 (ADOS-2; Lord et al., 2012) was employed to both confirm participants' ASD diagnoses and assess symptom severity. This instrument offers modules tailored to the child's chronological age and expressive language level. Each module generates a final score categorizing symptom severity as mild, moderate, or severe. The ADOS-2 is a semi-structured, standardized assessment tool regarded as the “gold standard” for ASD diagnosis and widely used in both clinical and research settings (Lord et al., 2012).

##### 2.2.3.2 Cognitive profile

To assess children's clinical profiles and mental age, we used different standardized tools based on the child's chronological age. We used the Griffiths Mental Development Scales (GMRS-Edition Revised, Luiz et al., 2006) for young children. These developmental scales assess various aspects of mental development in infants and children through semi-structured activities. The scales provide Z-scores across several subscales that yield a global quotient and developmental age equivalents. To assess older children's cognitive profile and mental age, we administered more structured standardized tests. According to child age, we used the Wechsler Preschool and Primary Scale of Intelligence (Freeman, 2021)–4th Edition, WIPPSI-4 with children until 6 years old; we used the Wechsler Intelligence Scale for Children, 4th Edition, WISC-IV with children from 6 to 16 years and 11 months; and Wechsler Adult Intelligence Scale–4th Edition, WAIS-IV for children older than 16 years.

### 2.3 Statistical analysis

First, descriptive statistics were performed. Next, the normality of variables was assessed using the Shapiro-Wilk test. Depending on the distribution, paired *t*-tests (for normally distributed variables) or Wilcoxon signed-rank tests (for non-normal distributions) were conducted to compare mothers and fathers taking into account variance dependence. To capture nuanced differences between mothers and fathers, both macro scales and subscales were included in the analysis. This approach allowed us to explore specific dimensions of parental stress (PSI-SF), mental health (SCL-90), and parent-reported child characteristics in greater detail (CBCL and SRS). Effect sizes were computed using paired Cohen's d for parametric tests (0.2 = small; 0.5 = medium; 0.8 = large), and rank biserial correlation for non-parametric paired wilcoxon tests (0.1 = small; 0.3 = medium; 0.5 = large). To control for Type I error increase due to multiple comparisons, the False Discovery Rate (FDR) correction was applied, using the Benjamini-Hochberg procedure (FDR = 0.05).

Linear Mixed Models (LMM) were employed to examine associations between parents' stress and mental health and their parent-reported perceptions of their children's behaviors and symptom severity. Additionally, we analyzed the association between parents' psychological dimensions and their clinically assessed children's cognitive functioning and symptom severity. Independent variables were standardized to account for their different numerical scales and to support model convergence. To account for the repeated measure nature of our data and to model the shared variance, the subject was included as a random intercept to account for individual variations. Moreover, child chronological age was always included as a covariate to account for the effect of age known in literature. The variable caregiver was also included as a predictor to account for caregiver differences. Ninety-five percentage confidence intervals were provided, and standardized coefficients were computed as an estimate of effect size for LMMs. Models were compared to baseline models including only the random intercept and child chronological age to derive a *p*-value for model significance. Model *p*-values were corrected using the Bonferroni method (*p*-limit = 0.05/5 = 0.01). Significant models' evaluation was performed considering marginal R2, AIC, and BIC with respect to baseline ones. Multicollinearity was assessed using the Variance Inflation Factor (VIF), considering VIF ≤ 3 as non-problematic. Finally, models were tested for assumptions.

A power analysis was conducted to determine the required sample size for detecting a medium effect size (Cohen's *d* = 0.4) in a paired-sample design using a two-tailed test with a significance level of α = 0.05 and power (1-β) = 0.80. For LLMs, power was estimated using the simr package (Green and MacLeod, 2016). We set the coefficient of the key predictor to match a standardized medium effect size of *d* = 0.4. The equivalent raw coefficient was computed as beta = *d* × total standard deviation of the outcome variable (combining residual and random effects) for each model. Power was computed based on 1,000 simulations and tested over numerical and categorical predictors. Power was >80% for all the simulated scenarios except for the effect of parent (power = 74%) and child chronological age (power = 78%) in the parental stress model. The analysis indicated that a minimum of 51 paired observations was required to achieve adequate statistical power. All analyses were implemented using R software 4.5 with the packages lme4, lmerTest, pwr, and effsize (R Core Team, 2021).

## 3 Results

### 3.1 Comparison between mothers and fathers

Considering parental stress, mothers and fathers did not differ significantly in their total stress levels, parental stress domain, dysfunctional parent-child interaction levels, or perception of difficult children (see Table 1).

**Table 1:** Mothers' and fathers' stress and mental health comparison with Wilcoxon signed-rank tests.

**Parent variables**	**Mother**		**Father**			
	***M***	**(** ***SD*** **)**	***M***	**(** ***SD*** **)**	**Adjusted** ***p*****-value**	**Effect size**
PSI-SF parental distress (PD)	63.25	31,4	52.02	26.89	0.06	0.28
PSI-SF difficult child (DC)	79.12	20.73	75.18	23.51	0.10	0.28
PSI-SF parent-child dysfunctional interaction (PDCI)	77.27	20.97	75.51	21.1	0.57	0.10
**PSI-SF total**	74.12	2,378	69.42	23.47	0.12	0.26
SCL 90-positive symptom total (PST)	37.63	20.66	23,24	18.14	< 0.001^***^	0.60
SCL 90-positive symptom distress index (PSDI)	1.71	0.53	1.4	0.40	< 0.01^**^	0.51
**SCL 90-global severity index (GSI)**	0.79	0.64	0.41	0.43	< 0.001^***^	0.58
Somatization	0.8	0.72	0.38	0.51	< 0.01^**^	0.51
Obsessive-compulsive	1.02	0.75	0.53	0.66	< 0.01^**^	0.57
Interpersonal sensibility	0.8	0.78	0.41	0.47	< 0.01^*^	0.58
Depression	1.1	0.89	0.47	0.55	< 0.001^***^	0.62
Anxiety	0.73	0.7	0.36	0.44	< 0.01^**^	0.45
Anger-hostility	0.64	0.63	0.42	0.58	0.04^*^	0.34
Phobic-anxiety	0.27	0.6	0.10	0.25	0.09	0.31
Paranoid ideation	0.86	0.75	0.57	0.67	< 0.05^*^	0.40
Psychoticism	0.45	0.57	0.26	0.42	< 0.01^**^	0.45

^*^p < 0.05.

^**^p < 0.01.

^***^p < 0.001.

Although no significant differences were observed in total stress levels, more prominent differences emerged regarding mental health. Mothers reported significantly higher levels on the Global Severity Index (GSI; *M* = 79; *SD* = 0.64) compared to fathers (*M* = 0.41; *SD* = 0.42; *W* = 1,106; *p* < 0.001). Similarly, mothers reported a higher number of symptoms (Positive Symptom Total, PST; *M* = 37.6 3; *SD* = 20.66) compared to fathers (*M* = 23.24; *SD* = 18.14; *W* = 1,075.5; *p* < 0.001) and greater distress associated with these symptoms (Positive Symptom Distress Index, PSDI; *M* = 1.71; *SD* = 0.53), compared to fathers (*M* = 1.40; *SD* = 0.40; *W* = 1,007.5; *p* = 0.001). Examining the subscales, mothers showed more prominently higher scores in depression (*M* = 1.1; *SD* = 0.9 vs. *M* = 0.47; *SD* = 0.55, *W* = 922.5; *p* < 0.001), in anxiety (M = 0.73; SD = 0.7 vs. M = 0.36; SD = 0.44, W = 891; p < 0.05), in obsessive-compulsive tendencies (*M* = 1.02; *SD* = 0.75 vs. *M* = 0.53; *SD* = 0.66, *W* = 714.5; *p* = *0.001*), and somatization (*M* = 0.8; *SD* = 0.72 vs. *M* = 0.38; *SD* = 0.51, *W* = 932; *p* = *0.001*, 0.05). The remaining subscale (phobic anxiety) revealed no significant results between mothers and fathers.

Regarding the parent-reported behavioral representation of children, we found that parents showed similar scores toward their children, revealing no significant differences in their perceptions of internalizing behaviors or externalizing behaviors (see Table 2). To conclude, regarding the perception of severity in their children's behavior, particularly in the area of socio-responsiveness, parents show no differences in their perceptions, displaying a consistent view of severity (see Table 2).

**Table 2:** Parent-reported child behaviors and symptoms severity.

**Parent-reported variables**	**Mother**		**Father**			
	**M**	**(SD)**	**M**	**(SD)**	**Adjusted** ***p*****-value**	**Effect size**
**CBCL-internalizing problems**	61.82	16.41	56.24	15.47	< 0.01^**^	0.54
**CBCL-externalizing problems** ^**a**^	54.73	10.7	50.88	9.38	< 0.01^**^	0.38
CBCL-withdrawal	65.57	11.49	62.22	10.11	< 0.05^*^	0.39
CBCL-anxiety/depression	63.43	11.55	58.59	10.41	< 0.01^**^	0.58
CBCL-somatic complaints	57.84	9.01	55.06	7.18	< 0.05^*^	0.59
CBCL-social problems	65.88	11.05	62.08	9.12	< 0.01^**^	0.46
CBCL-thought problems	64.86	10.02	62.08	9.33	< 0.05^*^	0.48
CBCL-attention problems	67,33	11.86	63.16	10.82	< 0.05^*^	0.42
CBCL-rule-breaking behavior	55.75	6.03	53.61	5.17	< 0.01^**^	0.60
CBCL-aggressive behavior	58.1	9.5	54.6	6.86	< 0.01^**^	0.65
SRS-social awareness^a^	66.13	14.88	62.63	13.98	0.06	0.24
SRS-aocial cognition	71	18.36	66.43	17.79	< 0.05^*^	0.33
SRS-social communication^a^	76.75	22.38	71.61	18.73	< 0.05^*^	0.24
SRS-social motivation^a^	75.14	19.35	70.2	17.30	< 0.05^*^	0.27
SRS-restricted interests and repetitive behavior	78.24	24.94	71.98	22.69	< 0.05^*^	0.39
**SRS-total**	78.65	21.59	72.94	19.17	< 0.05^*^	0.33

^*^p < 0.05.

^**^p < 0.01.

^***^p < 0.001.

^a^Paired T-tests with paired Cohen's d as effect sizes. The other tests were Wilcoxon signed-rank tests and used rank-biserial correlation as effect size measure.

### 3.2 Linear mixed models

Mixed models were employed to investigate the impact of parental mental health and perceived stress on their parent-reported representations of their children measured with the CBCL (for both internalizing and externalizing aspects) and SRS. Additionally, children's IQ and autism symptom severity (ADOS-2) were considered as dependent variables to provide a more comprehensive picture of the contribution of parental stress and mental health.

Children's internalizing behaviors, as perceived by their parents, were significantly associated with parent mental health, particulartly with the global severity index (*b* = 4.42; *t* = 3.06; *p* = 0.003; CI = [1.59, 7.25]; std *b* = 0.27). Model's intercept was also significant (*b* = 60.22; *t* = 29.41; *p* < 0.001; CI = [56.21, 64.24]; std *b* = 0.27). The other fixed effects predictors including parental stress (*b* = 1.20; *t* = 0.87; *p* = 0.38; CI = [−1.49, 3.88]; std *b* = 0.07), child chronological age (*b* = 1.48; *t* = 0.82; *p* = 0.42; CI = [−2.08, 5.01], std *b* = 0.09), and parent (*b* = −2.40; *t* = −1.77; *p* = 0.24; CI = [−6.38, 1.59]; std *b* = −0.15) were nonsignificant. Marginal *R*^2^ was 0.14 for the fixed effects. The model was significant when tested against the baseline model with child chronological age only (*X*^2^ = 20.65; *p* < 0.001). Information-based metrics confirmed the full model (AIC = 824; BIC = 843) goodness of fit with respect to the baseline one (AIC = 839; BIC = 849). The baseline model marginal *R*^2^ was 0.006. VIF was < 3 for all the model terms.

In contrast, children's externalizing behaviors were significantly associated with parental stress (*b* = 2.65; *t* = 3.06; *p* = 0.003; CI = [0.95, 4.35]; std *b* = 0.26). Model's intercept was also significant (*b* = 54.16; *t* = 42.50; *p* < 0.001; CI = [51.66, 56.65]; std *b* = 0.13). Parent (father) was also significant (*b* = −2.70; *t* = −2.08; *p* = 0.04; CI = [−5.25, −0.16]; std *b* = −0.27). The other fixed effects predictors including parental mental health (*b* = 0.91; *t* = 1.00; *p* = 0.32; CI = [−0.88, 2.70]; std *b* = 0.09) and child chronological age (*b* = 0.91; *t* = 0.81; *p* = 0.42; CI = [−1.28, 3.09]; std *b* = 0.09) were nonsignificant. Marginal *R*^2^ was 0.14 for the fixed effects. The model was significant when tested against the baseline model with child chronological age only (*X*^2^ = 22.00; *p* < 0.001). Information-based metrics confirmed the full model (AIC = 730; BIC = 748) goodness of fit with respect to the baseline one (AIC = 746; BIC = 756). The baseline model marginal *R*^2^ was 0.002. VIF was < 3 for all the model terms.

Children social responsiveness as perceived by their parents was significantly associated with parental stress (*b* = 4.76; *t* = 3.01; *p* = 0.004; CI = [1.66, 7.86]; std *b* = 0.23). Model's intercept was also significant (*b* = 77.18; *t* = 30.31; *p* < 0.001; CI = [72.19, 82.17]; std *b* = 0.07). The other fixed effects predictors including parental mental health (*b* = 2.98; *t* = 1.78; *p* = 0.08; CI = [−0.30, 6.26]; std *b* = 0.15), child chronological age (*b* = 2.83; *t* = 1.23; *p* = 0.22; CI = [−1.68, 7.35]; std *b* = 0.14), and parent (*b* = −2.77; *t* = −1.21; *p* = 0.23; CI = [−7.26, 1.73]; std *b* = −0.13) were nonsignificant. Marginal *R*^2^ was 0.14 for the fixed effects. The model was significant when tested against the baseline model with child chronological age only (*X*^2^ = 22.59; *p* < 0.001). Information-based metrics confirmed the full model (AIC = 861; BIC = 879) goodness of fit with respect to the baseline one (AIC = 877; BIC = 888). The baseline model marginal *R*^2^ was 0.009. VIF was < 3 for all the model terms.

Concerning child developmental quotient, the model was not significant (*X*^2^ = 2.62; *p* = 0.45). Information-based metrics also confirmed that the baseline model (AIC = 755; BIC = 766) better explained data instead of the full model (AIC = 759; BIC = 777). Finally, the model concerning autism severity did not converge and was therefore excluded.

## 4 Discussion

Given the extensive literature highlighting elevated stress and mental health challenges among parents of autistic children, particularly mothers (Enea and Rusu, 2020; Mello et al., 2022), this study aimed to investigate differences and associations in stress, mental health, parent-reported child, characteristics and clinically evaluated child clinical traits between Italian mothers and fathers of autistic children.

### 4.1 Comparison between parents

Our results revealed no differences between mothers' and fathers' total stress levels and its subdimensions. This aligns with prior research (McStay et al., 2014; Dunn et al., 2001) that underlines similar results between the two parents. Our results also seem to align with findings suggesting that caregiving an autistic child contributes to heightened stress levels in both parents. While our data do not directly assess paretnal involvement, previous research suggests that increased father engagement may help buffer maternal stress symptoms (Laxman et al., 2015). In fact, the increased involvement of fathers in society may balance caregiving roles, support mothers in their roles, and create a more cohesive family dynamic. This balance may reflect the dynamic interplay described by Family Systems Theory, where family members mutually influence one another, fostering equilibrium despite individual differences. Additionally, the Family Stress Model (Conger and Donnellan, 2007) suggests that shared caregiving responsibilities may reduce stressors that traditionally burden mothers disproportionately, such as daily caregiving strains. Although the parents'scores do not exceed the clinical cut-off, the mothers' scores are close to the threshold of significance. Considering parents' mental health, our findings indicate that mothers reported significantly higher severity levels of psychological symptoms, number of symptoms reported, and higher distress associated with those symptoms. In particular, when analyzing the subscales, our results suggest that mothers report higher levels of depression, anxiety, and obsessive and compulsive behaviors, consistent with previous research (Schnabel et al., 2020; Rattaz et al., 2023). In addition, mothers experienced higher levels of somatization compared to fathers (Zahl et al., 2024). It is interesting to note that the mean scores for mother depression exceed the clinical cut-off, indicating levels that require monitoring and attention due to potential depression, whereas the mean scores for fathers remain below the threshold. These mental health disparities suggest that maternal wellbeing may be particularly vulnerable, possibly due to role expectations. Consequently, women may feel overwhelmed when their experiences of motherhood do not match societal ideals, often neglecting their wellbeing to meet these expectations. Importantly, the mental health measure used in this study reflects individual psychological traits rather than caregiving roles, reinforcing the need for tailored interventions that address personal vulnerabilities in addition to parental responsibilities. Taken together, this role-based divergence underscores the importance of involving both parents in comprehensive evaluations to capture a holistic picture of the child's experiences (Piro-Gambetti et al., 2024).

With regard to parent-reported child behaviors and autistic symptoms, we found that mothers reported significantly higher levels of internalizing and externalizing problems, as well as greater severity of autism-related traits, compared to fathers. This is in line with previous studies that suggested that mothers tend to report higher levels of internalizing problems in their children (Piro-Gambetti et al., 2024), potentially because they are more exposed to them, although findings remain inconclusive (e.g., van der Veen-Mulders et al., 2018). It is also noteworthy that, on average, mothers in this study reported higher levels of mental health issues than fathers, which may have shaped their perceptions and led to an overestimation of their child's internalizing and externalizing behaviors, as well as their severity in the socio-responsive domain. Interestingly, although mothers reported higher levels of perceived autistic symptomatology, especially in manner ism and social communication, there are no differences between mothers and fathers considering the child awareness. As the ability to pick up social cues, the child awareness, seem to be perceived as less severe by both parents and locates in the mild range of severity, whereas the other scales consistently locate in the moderate or highly intense severity despite parental differences.

### 4.2 Associations between parents and parent-reported child characteristics

Our study provides new insights into the complex associations between parental psychological wellbeing and perceived child functioning in families of autistic children.

Regarding internalizing behaviors, our findings show a significant association with parental mental health. This supports existing literature suggesting that caregivers experiencing greater psychological distress may be more sensitive to, or perhaps more likely to perceive, internalizing difficulties in their children (Choi et al., 2023). Notably, neither perceived stress, parent gender, nor child age significantly predicted internalizing problems in this model. This suggests that the global burden of psychological symptoms in parents, rather than context-specific parenting stress, may play a more prominent role in shaping how internal struggles in the child are perceived and reported.

Interestingly, child externalizing behaviors were significantly associated with parental stress, but not by parents' general mental health. These findings align with previous research showing that overt and disruptive behaviors are particularly demanding for caregivers and often elicit reactive stress responses (Rodriguez et al., 2019; Ooi et al., 2016). In addition to this, our results did not reveal significant effects of parent gender on internalizing outcomes only, suggesting that the associations identified were consistent across mothers and fathers for internalizing aspects. Conversely, fathers tended to have a more positive representation of their children's externalizing behaviors even when considering parental stress, mental health, and child chronological age. Notably, as discussed earlier, when analyzing parent-reported CBCL and SRS scores separately, mothers reported higher levels of both behavioral problems and autistic traits compared to fathers. This apparent contradiction may indeed reflect the specific influences of different variables on parental perception of autistic children. In fact, our analysis revealed that the general effect of parent remained significant only for externalizing behaviors, disappearing when including parental stress and mental health in regression models. Therefore, mothers' and fathers' differences in perceiving their children may be mediated by these psychological factors that should be therefore specifically taken into account during clinical care.

In addition to behavioral outcomes, we also explored whether parental psychological traits were associated with more objective indicators of child functioning, including cognitive abilities and clinician-rated autism severity. No significant associations emerged between parental stress or mental health and the child's cognitive functioning, as measured by standardized IQ assessments. Furthermore, we were unable to analyze associations with the ADOS score due to convergence issues in the model estimation process, probably associated with the ordinal nature of the ADOS-2 calibrated severity score that does not align with linear regression. Taken together, our results suggest that different aspects of parental psychological functioning may be differentially associated with child behavioral outcomes, depending on whether these behaviors are internalizing or externalizing in nature. These findings support a personalized approach to families, emphasizing the importance of evaluating both parental mental health and parenting-related stress, and incorporating multiple assessment sources to capture child functioning.

### 4.3 Implications

The findings of this study underscore the need for differentiated support strategies tailored to the unique psychological needs of mothers and fathers while fostering collaborative approaches. Mothers may benefit from targeted support addressing internalizing symptoms such as depression and anxiety, emphasizing coping strategies and mental health support. Research within families is essential for understanding how to best support those living with ASD. Moreover, despite preliminary studies highlighting the unique role of family support in shaping family functioning, limited research has explored its specific influence (Blackledge and Hayes, 2006; Gavidia-Payne et al., 2015; Hastings, 2003; Rao and Beidel, 2009). Targeted interventions could be particularly impactful, as frequent exposure to parental depressive symptoms and recurrent or severe episodes of parental depression are linked to an increased risk of mental health problems in children. This association is especially pronounced for internalizing issues (e.g., mood disorders) in both neurotypical children (Kamins, 2021; Schnabel et al., 2020; Tirumalaraju et al., 2020) and autistic children (Cohen and Tsiouris, 2006; Wiggins et al., 2019). Parents experiencing depression often struggle to provide sensitive and responsive caregiving, which can further exacerbate risks for adverse child outcomes (Piro-Gambetti et al., 2024; Taraban and Shaw, 2018).

Parenting programs should also foster shared strategies for managing stress related to externalizing behaviors, creating a unified approach to addressing disruptive behaviors in children. From a broader perspective, clinicians and educators should recognize the cultural and specific roles that shape parental experiences. By tailoring interventions to respect these nuances, practitioners can enhance both parental wellbeing and child developmental outcomes. Furthermore, these findings emphasize the importance of including fathers in research and interventions, as their unique perspectives contribute significantly to family dynamics.

This study highlights the distinct and shared challenges faced by mothers and fathers of autistic children, providing valuable insights for developing targeted, evidence-based interventions. By addressing the unique psychological needs of each parent while fostering collaborative approaches, interventions can promote better outcomes for both parents and children. Future research should continue exploring these dynamics across diverse cultural contexts to enhance the applicability and impact of these recommendations as well as exploring the bidirectional mechanisms linking parent and child functioning.

### 4.4 Limitations

This study has several limitations that warrant further investigation. First, althought the sample size was statistically adequate based on power calculations, the limited sociodemographic diversity of the sample may constrain the generalizability of the findings. the small sample size may require additional data to confirm findings and allow for generalization. Second, the study did not account for contextual elements such as external social support, time dedicated to caregiving activities, and socioeconomic factors, which may moderate the relationship between parental stress, mental health, and child characteristics. Future research should explore how marital relationships, parenting practices, mothers and fathers' relationships (and roles), and available support resources impact these associations. Third, while the inclusion of both parent-reported and clinician-rated measures was a strength, the internalizing and externalizing child behaviors domains were assessed only through parent report.

Additionally, the presence or absence of siblings was not considered, which may play a role in family dynamics and parental stress. Examining these factors could provide a more comprehensive understanding of the challenges and resilience factors in families with autistic children. To conclude, future research should also focus on considering parents' personality traits and their associations and/or moderation role in the relationship between stress and mental health and child characteristics.

### 4.5 Conclusion

In conclusion, this study offers insights into the psychological wellbeing of mothers and fathers raising autistic children, highlighting both shared and distinct patterns in their experiences. By combining parental and clinical based assessments, we found how stress and mental health symptoms are differentially associated with parent-reported perceptions of child functioning. These findings underscore the importance of adopting a dyadic approach that includes both caregivers in assessment and intervention practices.

## Data Availability

The raw data supporting the conclusions of this article will be made available by the authors, without undue reservation.
